# Attenuation of Typical Sex Differences in 800 Adults with Autism vs. 3,900 Controls

**DOI:** 10.1371/journal.pone.0102251

**Published:** 2014-07-16

**Authors:** Simon Baron-Cohen, Sarah Cassidy, Bonnie Auyeung, Carrie Allison, Maryam Achoukhi, Sarah Robertson, Alexa Pohl, Meng-Chuan Lai

**Affiliations:** 1 Autism Research Centre, Department of Psychiatry, University of Cambridge, Cambridge, United Kingdom; 2 Cambridge Lifespan Asperger Syndrome Service (CLASS) Clinic, Cambridgeshire and Peterborough National Health Service Foundation Trust, Cambridge, United Kingdom; 3 Department of Psychology, University of Edinburgh, Edinburgh, United Kingdom; 4 Department of Psychiatry, National Taiwan University Hospital and College of Medicine, Taipei, Taiwan; The George Washington University, United States of America

## Abstract

Sex differences have been reported in autistic traits and systemizing (male advantage), and empathizing (female advantage) among typically developing individuals. In individuals with autism, these cognitive-behavioural profiles correspond to predictions from the “extreme male brain” (EMB) theory of autism (extreme scores on autistic traits and systemizing, below average on empathizing). Sex differences within autism, however, have been under-investigated. Here we show in 811 adults (454 females) with autism and 3,906 age-matched typical control adults (2,562 females) who completed the Empathy Quotient (EQ), the Systemizing Quotient-Revised (SQ-R), and the Autism Spectrum Quotient (AQ), that typical females on average scored higher on the EQ, typical males scored higher on the SQ-R and AQ, and both males and females with autism showed a shift toward the extreme of the “male profile” on these measures and in the distribution of “brain types” (the discrepancy between standardized EQ and SQ-R scores). Further, normative sex differences are attenuated but not abolished in adults with autism. The findings provide strong support for the EMB theory of autism, and highlight differences between males and females with autism.

## Introduction

Typical males on average score higher on measures of autistic traits (i.e., the individual features that comprise the quantitative variation in domains of cognition and behaviour associated with autism) [1] than do typical females [2]–[5]. In addition, the sex ratio of the prevalence for autism spectrum conditions (henceforth ‘autism’) is male-biased [6]. The *extreme male brain* (EMB) theory of autism explains these two findings by positing that there are typical male and female cognitive profiles (‘brain types’) in the general population, in two domains: empathizing (the drive and ability to identify a person's thoughts and feelings, and to respond to these with an appropriate emotion) [7] and systemizing (the drive and ability to analyse or build systems) [8]. Typical females, on average, exhibit more empathizing and less systemizing compared to typical males, and people with autism show an extreme of this ‘male profile’ [9], [10].

One unresolved issue is whether normative sex differences in this cognitive profile in the general population are also observed in autism. This is important for identifying sex-specific autism phenotypes [11], understanding the biological basis of these phenotypes [12], [13], and deepening our understanding of females with autism [9]. The few studies investigating cognitive and behavioural sex differences within autism show inconsistent results: some studies have found no significant sex differences in autism [14], [15], whilst other studies have found some sex differences, on a mixed set of measures [16]–[21]. For example, a meta-analysis (based on smaller-scale studies) [22] and large-scale studies [20], [23] indicate that females show fewer repetitive, restricted behaviours and interests. Cognitively, most differences between males and females with autism (when compared respectively to neurotypical males and females) are observed in executive functions and visuospatial processing, whereas they tend to share similar levels of social-emotional cognitive difficulties [18], [19], [24]. It is unclear what the relevance of these is to the EMB theory as normative sex differences were not always tested, and these tasks may not load on to empathizing or systemizing. One study found females with autism had slightly but significantly higher scores on the Autism Spectrum Quotient (AQ) via self-report, even though they scored lower than males with autism on the Autism Diagnostic Observation Schedule (ADOS) [21]. This suggests they may have developed strategies to better ‘camouflage’ their social-communication difficulties. Overall, it remains unclear if autism abolishes or simply attenuates normative sex differences.

Most previous studies also suffer from relatively small sample sizes. An exception is the largest study to date using the Simons Simplex Collection, comparing autism symptoms in 304 female and 2,114 male children with ‘simplex’ autism, aged 4–18 years [25]. This study found that, compared to males, females have somewhat greater social communication impairments, fewer restricted interests (but not fewer repetitive behaviours and stereotypies), poorer adaptive skills, and higher level of externalizing problems. There is, however, no study with a comparative sample size in adults with autism. In many of the above studies there is a relative under-representation of females with autism, especially those with average or above-average intelligence [22]. Small and sex-unbalanced sample sizes may affect the statistical power to detect sex differences with small or medium effect sizes [26]. In addition, comparing studies testing very young children [17] with others including a broad age range [16] may obscure sex differences due to developmental changes. Furthermore, sex differences within autism may vary with IQ, and whilst some studies have matched for age and IQ, others have not. Previous studies have also varied in whether measures were retrospective parent-reports [16] or direct observation [17], and this could produce discrepant results [27], [28].

To overcome these confounds, and to test the EMB theory directly, Lai et al. tested 33 male and 29 female adults with high-functioning autism or Asperger syndrome, matched for age and IQ, and found no sex differences on the EQ or SQ-R [21]. This suggests that normative sex differences on these measures are abolished in autism [7], [8]. Wheelwright et al. investigated 69 males and 56 females with high functioning autism or Asperger syndrome, and also found typical sex differences on self-report scores of EQ or SQ-R were abolished in autism [29]. Auyeung et al. studied 46 girls and 219 boys with autism, and they too confirmed that typical sex differences in parent-reported empathizing and systemizing were abolished in autism [30]. These findings from studies of empathizing and systemizing are consistent with the EMB theory of autism [10].

In the present study, we attempt to overcome the issue of statistical power by investigating sex differences in the largest, and importantly, most sex-balanced sample in adults to date: over 800 individuals with autism. Again we focus on empathizing and systemizing because these have given the clearest results to date, both in terms of normative sex differences, and the predicted attenuation or absence of these sex differences in autism, based on the EMB theory. We selected individuals over 18 years of age since dispositional traits of empathy and systemizing, such as aspects of personality, are likely to be stabilized by adulthood. In addition, all individuals were high-functioning, so that sex differences could be investigated independent of learning disability. We used online self-report questionnaires to gather a very large sample, which increased statistical power in detecting sex differences, even if these were attenuated in autism.

## Method

### Participants

Participants were recruited online. After exclusions (see below), the autism group comprised 811 individuals (454 females, 357 males) who completed questionnaires at one of two websites (www.autismresearchcentre.com or www.cambridgepsychology.com) and reported having a formal clinical diagnosis of an autism spectrum condition. Diagnoses comprised Asperger Syndrome (*n* = 506), High Functioning Autism (*n* = 41), Autism (*n* = 11), Pervasive Developmental Disorder (*n* = 15), and Autism Spectrum Condition (participants who did not specify a subtype) (*n* = 238).

After exclusions (see below), the typical control group comprised 3,906 individuals (2,562 females, 1,344 males), who completed questionnaires and who reported they had no diagnosis of an autism spectrum condition, via www.cambridgepsychology.com. Anyone from this group who reported having a child or other family members with autism was excluded from the control group, to avoid inadvertently including those with the ‘broader autism phenotype’ [31]. Individuals with a diagnosis of bipolar disorder, epilepsy, schizophrenia, attention-deficit/hyperactivity disorder (ADHD), obsessive-compulsive disorder (OCD), learning disability (LD), an intersex/transsexual condition, or psychosis were excluded from both groups. Outliers, defined as having a z-score of 3.29 or greater on each measure, were also excluded.

Participants were aged between 18 and 75 years old (see Table 1) and those in the autism group did not differ in age from the control group (F(1, 4713) = .21, *p* = .63). A majority of the individuals in the autism group provided information on type of education (mainstream, home, special) (n = 769), and of these individuals a majority reported having attended mainstream school (n = 679; 88.3%). A majority of the autism group also provided information on current occupation (n = 676), and of these a majority (n = 471; 69.7%) were currently employed, 115 (17%) individuals were in full time study, and 90 (13.3%) individuals were unemployed. In the control group, 1,709 individuals provided information on their education type, and of these 1,679 (98.2%) individuals reported having mainstream education. In total 2,648 control individuals provided information on their occupation, and of these 2,184 (82.5%) were currently employed, 424 (16%) were in full time study and 40 (1.5%) were unemployed.

**Table 1:** Mean scores for each measure (with standard deviations, SDs).

Group	Sex		Age	AQ	EQ	SQ-R	D
**Autism**	Female	Mean	34.5	32.9	26.4	71.7	0.15
		SD	13.1	11.5	17.2	25.7	0.17
		n	454	454	419	397	397
	Male	Mean	34.9	34.8	20.4	78.4	0.22
		SD	13.3	9.1	12.4	24.3	0.12
		n	357	357	307	295	295
**Control**	Female	Mean	34.4	17.1	48.5	55.1	−0.04
		SD	12.5	7.6	13.7	21.1	0.12
		n	2562	2562	2376	2261	2261
	Male	Mean	34.4	20.3	38	68.1	0.07
		SD	14.3	7.8	13.7	21.6	0.11
		n	1344	1344	1253	1197	1197

### Ethical Approval

Ethical approval was from the Psychology Research Ethics Committee (PREC), University of Cambridge, UK. There is no reason to question if adults with Asperger Syndrome (AS) or High-Functioning Autism (HFA) can give informed consent since by definition they have at least average, if not above-average IQ, and have normal intellectual competence. Consent was obtained online when participants registered to join the research database and where they had the opportunity to read the Terms and Conditions, which included how their data will be used for research and how their personal information is only seen by named database managers who take legal responsibility for data protection. This data covers both their questionnaire data but also performance data each participant provides, and their willingness to be re-contacted to hear about new studies. This consent procedure was approved by PREC as well.

### Measures

We used the following four measures: (1) The Empathy Quotient (EQ) quantifies individual differences in empathizing [7]. (2) The Systemizing Quotient-Revised (SQ-R) [29] measures individual differences in systemizing [8]. (3) The Autism Spectrum Quotient (AQ) measures the degree to which an adult with an average or above-average IQ has autistic traits [2]. (4) D score/‘Brain Type’ is a measure of the standardized difference between an individual's empathizing and systemizing scores [30], [32]. The raw SQ-R and EQ scores are standardized by subtracting the typical population mean (denoted by <…>) from the participant's score and then dividing this by the maximum possible score (S = (SQ-R–<SQ-R>)/150 and E = (EQ–<EQ>)/80). The control group means are used as estimations of the typical population means in this standardization procedure: EQ (*mean*  = 44.87, *SD* = 14.58) and SQ-R (*mean*  = 59.66, *SD*  = 22.15). The difference (D) between the standardized EQ and SQ-R scores is then calculated by: D = (S–E)/2. Using the D score, individuals can be classed into one of five cognitive profiles, or ‘brain types’. ‘Brain types’ based on D score are quantitatively defined in Table 2, based on a prior study [32] which classed the lowest and highest 2.5^th^ percentiles of scores in a large population-based typically developing group as ‘Extreme Type E’ (E>>S) and ‘Extreme Type S’ (S>>E) respectively. Those scoring between the 2.5^th^ and 35^th^ percentiles are classed as ‘Type E’ (E>S), those between the 35^th^ and 65^th^ percentile as ‘Type B’ (balanced, E≈S), and those between the 65^th^ and 97.5^th^ percentile as ‘Type S’ (S>E).

**Table 2:** Brain type boundaries based on the D score, calculated from the current sample and a previous population-based adult sample.

Brain type	Brain type boundary	Brain type boundary^a^
Extreme E	**D**<−0.23	**D**<−0.21
Type E	−0.23≤**D**<−0.053	−0.21≤**D**<−0.041
Type B	−0.053≤**D**<0.048	−0.041≤**D**<0.040
Type S	0.048≤**D<**0.277	0.040≤**D<**0.21
Extreme S	**D**≥0.277	**D**≥0.21

a Data from [29].

### Statistical Analysis

Large samples increase the robustness of ANOVA to violation of normality and homogeneity of variance. Separate two-way ANOVAs were conducted on AQ, EQ, SQ-R and D, with two between-subject factors of ‘Diagnosis’ (autism vs. control) and ‘Sex’ (female vs. male). Sex-by-diagnosis interaction effects indicate whether the effect of sex is dependent on the diagnostic status. Significant interaction effects are followed up by simple main effects analysis to establish whether significant sex differences exist in each diagnostic group and to reveal whether the interaction is ordinal or disordinal. Effect sizes were calculated using omega (ω) for main effects and interactions and Cohen's *d* for focused comparisons (simple main effects). For calculation of omega, the harmonic mean sample size (the average sample size) is used to correct for unequal sample size between groups. Omega has the same benchmarks for effect size as *r*: 0.1 =  small effect, 0.3 =  medium effect and 0.5 =  large effect. As for Cohen's *d*: 0.2 =  small effect, 0.5 =  medium effect and >0.8 =  large effect.

## Results

**Figure 1:**
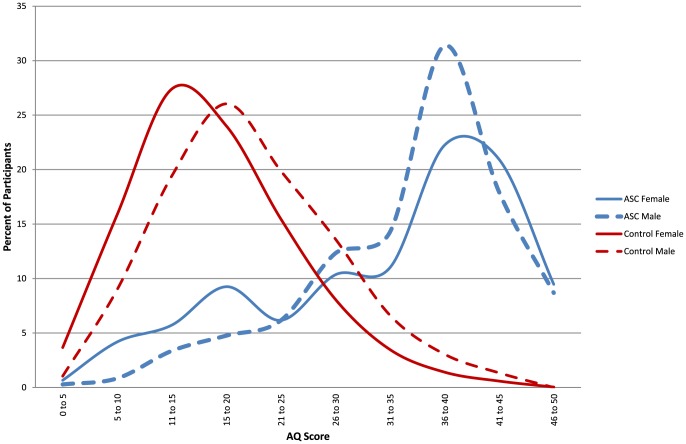
The distributions of Autism Spectrum Quotient (AQ) scores by the four groups: males and females with and without autism spectrum conditions.

**Figure 2:**
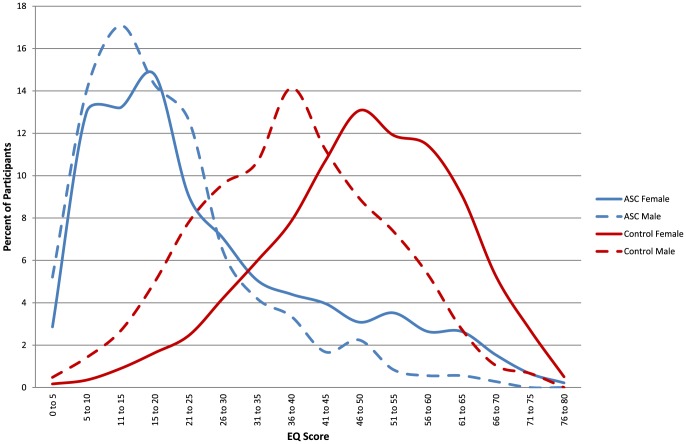
The distributions of Empathy Quotient (EQ) scores by the four groups: males and females with and without autism spectrum conditions.

**Figure 3:**
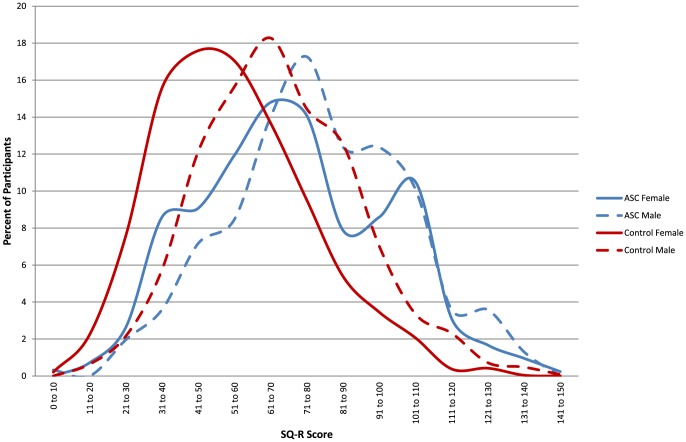
The distributions of Systemizing Quotient-Revised (SQ-R) scores by the four groups: males and females with and without autism spectrum conditions.

Table 1 shows the mean scores for AQ, EQ, SQ-R and D for males and females in the autism and control groups. Figures 1–3 show the distribution of scores for AQ, EQ and SQ-R in the control and autism male and female groups.

### AQ

An ANOVA revealed significant main effects of Diagnosis (F(1, 4713) = 2207, *p*<.001, ω = .67) and Sex (F(1, 4713) = 63, *p*<.001, ω = .11). AQ scores were higher in the autism group than the control group, and higher in males than females. There was a significant ordinal interaction between Sex and Diagnosis (F(1, 4713) = 3.94, *p* = .047, ω = .02), reflecting that sex differences were smaller in the autism than the control groups. Simple main effect analysis showed that typical males scored significantly higher than typical females (F(1, 4713) = 133, *p*<.001, *d* = .41), and males with autism scored significantly higher than females with autism, though with a small effect size (F(1, 4713) = 10.87, *p*<.001, *d* = .18). This indicates that normative sex differences were attenuated, but not absent in the autism group.

### EQ

An ANOVA revealed significant main effects of Diagnosis (F(1, 4351) = 1171.5, *p*<.001, ω = .56) and Sex (F(1, 4351) = 202.6, *p*<.001, ω = .23). EQ scores were lower in the autism group than the control group, and higher in females than males. There was also a significant ordinal interaction between Sex and Diagnosis (F(1, 4351) = 14, *p*<.001, ω = .06), again reflecting that sex differences were smaller in the autism than the control groups. Simple main effect analysis showed that typical females scored significantly higher than typical males (F(1, 4351) = 455, *p*<.001, *d* = .76), and females with autism scored significantly higher than males with autism, though with a relatively smaller effect size (F(1, 4351) = 33.4, *p*<001, *d* = .40). This also indicates that normative sex differences were attenuated, but not absent in the autism group.

### SQ-R

An ANOVA revealed significant main effects of Diagnosis (F(1, 4146) = 206.87, *p*<.001, ω = .28) and Sex (F(1, 4146) = 11.97, *p*<.001, ω = .21). SQ-R scores were higher in the autism group than the control group, and higher in males than females. There was also a significant ordinal interaction between Sex and Diagnosis (F(1, 4146) = 11.6, *p*<.001, ω = .06), once again reflecting that sex differences were smaller in the autism than the control groups. Simple main effect analysis showed that males scored significantly higher than females in the control group (F(1, 4146) = 275.36, *p*<.001, *d* = .61), and in the autism group but to a lesser extent (F(1, 4146) = 15.6, *p*<.001, *d* = .27). This also indicates that the normative sex difference was attenuated, but not absent in the autism group.

### D-score and Brain Types

**Figure 4:**
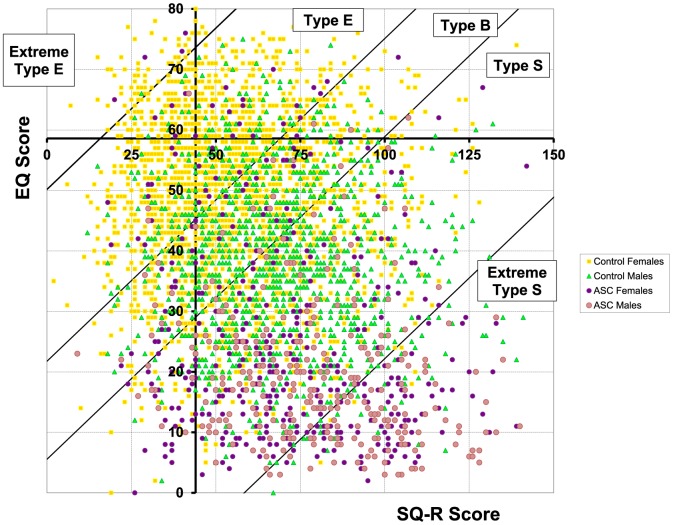
The distributions of males and females with and without autism spectrum conditions by ‘brain type’ (or cognitive style), in relation to their EQ and SQ-R scores.

Table 2 shows the brain type boundaries calculated from the current sample and a previous population-based adult sample [29]. Table 3 shows the percentage of participants with each brain type by group. In the control group more males than females were in Type S and Extreme Type S, and more females than males were in Type E and Extreme Type E. In the autism group, there was a shift towards Type S and Extreme Type S for both males and females, and there were more females than males with autism in Type B, Type E and Extreme Type E. This is shown in Figure 4, in which the D axis runs from the top left corner to the bottom right corner. It is clear that typical females cluster in the top left corner with the lowest D scores, followed by typical males, followed by females with autism, and finally by males with autism in the bottom right corner, and with the highest D scores. Males and females with autism appear to have a greater scatter across the brain types than the typical control groups.

**Table 3:** Percentage of participants with each brain type in the present study, listed alongside a previous population-based study.

Brain Type	Autism Females (n = 397)	Autism Males (n = 295)	Control Females (n = 2,261)	Control Males (n = 1,197)	Autism Group^a^ (n = 125)	Control Females^a^ (n = 1,038)	Control Males^a^ (n = 723)
Extreme E	1.5	0	3.7	0.3	0	4.3	0.1
Type E	11.6	1.0	43.1	12.9	0	44.8	15.1
Type B	12.6	7.4	30.6	28.4	6.4	29.3	30.3
Type S	46.8	60.3	21.2	53.7	32	20.7	49.5
Extreme S	27.4	31.3	1.4	4.7	61.6	0.9	5

a Data from [29].

These findings were supported by an ANOVA on the D score, which revealed significant main effects of Diagnosis (F(1, 4146) = 1058.8, *p*<.001, ω = .55) and Sex (F(1, 4146) = 259.68, *p*<.001, ω = .27). D scores were higher in the autism group than the control group, and higher in males than females. There was a significant ordinal interaction between Sex and Diagnosis (F(1, 4146) = 21.59, *p*<.001, ω = .07), demonstrating that sex differences were smaller in the autism than the control group. Simple main effects analysis showed that males had significantly higher D scores than females in the control group (F(1, 4146) = 606, *p*<.001, *d* = .95), and also in the autism group but to a lesser extent (F(1, 4146) = 39.99, *p*<.001, *d* = .41). Again this indicates that normative sex differences were attenuated, but not absent, in the autism group.

Table 4 shows a comparison of demographic characteristics between individuals with two opposite sets of brain types (‘Extreme Type S and Type S’ vs. ‘Extreme Type E, Type E and Type B’) in the male and female autism groups. In the male autism group, individuals with ‘Extreme Type E, Type E and Type B’ brain types were significantly lower in mean age (*t*(293) = 2.6, *p* = .01), and were significantly more likely to be in full time education (odds ratio OR = 3.66, 95% confidence interval CI = [1.28, 10.42]; χ^2^(1) = 6.6, *p* = .01), than individuals with ‘Extreme Type S and Type S’ brain types. In the female autism group, individuals with ‘Extreme Type E, Type E and Type B’ brain types were significantly more likely to be employed (OR = 2.1, 95% CI = [1.1, 3.96]; χ^2^(1) = 5.4, *p* = .02), significantly less likely to be unemployed (OR = 0.25, 95% CI = [0.057, 1.08]; χ^2^(1) = 3.97, *p* = .046), and marginally significantly more likely to have had a mainstream education (OR = 2.45, 95% CI = [0.93, 6.48]; χ^2^(1) = 3.49, *p* = .06), than individuals with ‘Extreme Type S and Type S’ brain types.

**Table 4:** Comparison of demographic characteristics between the two opposite sets of brain types in the male and female autism groups.

Demographic Characteristics	Autism Females		Autism Males	
	Extreme Type E, Type E and Type B	Extreme Type S and Type S	Extreme Type E, Type E and Type B	Extreme Type S and Type S
**Mean Age** (n)	36.8 (102)	33.7 (295)	29.4** (25)	36** (270)
**Employment Status**	***n = 84***	***n = 247***	***n = 20***	***n = 229***
% Employed	83.33*	70.44*	60	67.69
% Unemployed	2.39*	8.91*	10	21.83
% In full time education	14.28	20.65	30**	10.48**
**Education Type**	***n*** ** = 98**	***n*** ** = 283**	***n*** ** = 25**	***n*** ** = 220**
% Mainstream education	94.8	88.34	80	88.6

*p<.05,

**p<.01.

## Discussion

Consistent with previous smaller-scale studies [8], [29], [30] we confirmed that in a very large typical control group, females on average score higher on EQ, males on average scored higher on AQ and SQ-R, and that both males and females with autism show a shift to the extreme of the ‘male profile’. Using EQ and SQ-R scores to calculate D score, which corresponds to specific cognitive ‘brain types’, Type E was the most frequent profile amongst typical females, Type S was the most frequent profile in typical males, and Type S and the Extreme Type S were the most common ones in the autism group for both males and females. A diagnosis of autism shifted the profiles of both males and females towards the ‘extreme-male’ end (indicated by the same direction of significant main effects of Diagnosis and Sex in the ANOVAs), and the patterns of normative sex differences were significantly attenuated in individuals with autism (indicated by the significant ordinal interaction between Sex and Diagnosis). These findings fit the predictions from the EMB theory of autism [10]. This likely reflects a more pronounced effect of ‘masculinization’ (i.e., shifting towards the typical male-end of the profile) in females with autism in these aspects. This is also in line with recent neuroimaging studies showing ‘masculinization’ of the brain in females with autism [12], [33], [34] and likely reflects sex-linked biological factors at work in autism, such as foetal testosterone [35], [36].

Data from this large study also provide an adequately powered test of whether normative sex differences in autistic traits, empathizing, systemizing and cognitive ‘brain type’, previously documented in typical individuals, are also present in individuals with autism. Although the patterns of normative sex differences were attenuated in individuals with autism, significant sex differences were still evident, in the same direction as in the control group (indicated by the significant ordinal Sex-by-Diagnosis interaction and the significant differences shown by simple effect analyses). The fact that significant sex differences within autism were found in the present study is likely due to the substantially larger sample size (6.5 times larger than the largest of previous studies [29]), which provides greater power to detect small and medium effect size sex differences.

The persistence of normative sex differences in autism, found in this high-functioning adult cohort that is the largest to date, is notable and fits with recent reflections about what the field may have missed regarding females with autism. First, even though both sexes have been clinically diagnosed with autism, autism-related trait characteristics in males and females with autism distribute differently on average. These traits, continuously distributed in the general population, have well-established sex-differential distributions [2], [7], [8]. Therefore, our finding here echoes the call to consider sex-differential thresholds for clinically diagnosing autism [1], [37]. Second, the persistence of normative sex differences in autism corresponds with recent findings of less male-typical, possibly ‘compensated’, ‘masked’ or qualitatively different ‘female phenotypes’ of autism [18], [20], [21], [25], [38], [39]. For example, in our sample there were a moderate proportion of females with autism having a Type B, Type E or even an Extreme Type E profile (25.7%, who were also more likely to be employed and had received mainstream education compared to those in Type S or Extreme Type S), in contrast to males with autism (8.4%), suggesting that there is substantial variability in ‘brain type’ profiles in this group. A less male-typical presentation of autism in females, especially in high-functioning individuals, may be related to the existing diagnostic bias towards males [39]–[41]. Further studies are needed to directly address the various presentations of autism in females and how they are similar to or different from those of males.

This study has several limitations. First, only individuals who were capable of self-reporting formal clinical diagnosis of an autism spectrum condition were investigated, so the observed sex differences may not generalize to subgroups with intellectual disability or with substantial communication difficulties. Generalizability could be tested via parent-report of children on the autistic spectrum, irrespective of the individual's age or IQ. Second, since the data were all collected online, it is unknown if the findings would generalize to individuals who cannot access the internet to volunteer for research. Third, individuals with certain co-occurring conditions or major psychiatric conditions were excluded, so it is unknown how these might modulate cognitive profiles. Fourth, there was no independent verification of diagnosis for the majority of the autism group since participants were recruited online. However, this approach did allow us to obtain a much larger sample than could otherwise have been investigated. Previous studies have shown high levels of agreement between self/parent-reported and clinician-reported diagnosis [42], and all individuals with autism in the present study provided the name of the clinician and the clinic where they were diagnosed, so there is no obvious reason to question their diagnoses. In addition, a subset (n = 64) attended the National Health Service Cambridge Lifespan Asperger Syndrome Service (CLASS) clinic in Cambridge, where diagnosis was independently confirmed in person.

Although there is a long-held view that the male-bias in prevalence is particularly extreme in the high-functioning end of the autism spectrum (e.g., up to 14∶1 [43]), recent large-scale epidemiological data indicate that the sex ratio at the high-functioning end is not as extreme as previously believed, but rather falling between 2–5∶1 [44]–[48]. This suggests that the earlier view may be due to under-recognition of high-functioning females in the past. With increased awareness and improved clinical recognition, increasing numbers of high-functioning females with autism volunteer for research to help our understanding of how autism manifests in females. This is probably one reason why, in the present study, we were able to recruit even more females than males with autism. Although this sex ratio may not be representative of the autism population at large, the comparable sample size of males and females with autism in the present study is desirable for a statistically robust investigation of sex differences within autism (which has not been attainable in earlier studies).

The growing evidence of ‘masculinization’ in females with autism now covers five different levels: behaviour, medical symptomatology, cognition, neural, and endocrine. At the behavioural level females with autism are shifted along the continuum from ‘typical females’ to ‘typical males’ with regard to gender-stereotyped behaviours [49]–[51]. At the medical symptomatology level, females with autism show higher rates of testosterone-driven conditions such as Polycystic Ovary Syndrome (PCOS) and severe acne [49], [52]. At the cognitive level, females with autism are shifted beyond the ‘male-end’ along the ‘typical females’—‘typical males’ continuum on the AQ, EQ, SQ-R (present data), and the Reading the Mind in the Eyes test (Baron-Cohen et al., in preparation). At the neural level, females with autism show a shift towards ‘masculinization’ in both grey and white matter brain morphology [12]. Finally, at the endocrine level, females with autism show elevated serum levels of androgens [53]–[55]. On the other hand, a recent large-scale population-based study shows that elevated levels *in utero* of all of the Δ4 sex steroid pathway (progesterone, 17α-hydroxy-progesterone, androstenedione, and testosterone), as well as cortisol, predict later diagnosis of autism in males [56]. It will be important to now test this in females who go on to develop autism, in order to understand early plausible developmental mechanisms associated with later ‘masculinization’ across multiple levels.

We conclude that when measuring empathizing, systemizing, and autistic traits in a large, adequately powered sample of high-functioning adults with autism via self-report, results provide strong support for predictions from the EMB theory [10] that the cognitive profiles of both males and females with autism are shifted towards and beyond the typical male-distribution, and normative sex differences in these profiles are attenuated in autism. However, significant sex differences within autism (with small to medium effect sizes) are evident, indicating a persistence of normative sex differences despite the clinical diagnoses of autism. Future research needs to address what factors (e.g. prenatal hormonal effects, sex-linked genetic and epigenetic factors, and other mechanisms associated with the regulation of gene expression) [57], [58] contribute to the emergence of the cognitive ‘masculinization’ in autism [9], and characterize the similarities and differences between males and females with autism [12], [13], [39] to help clarify the substantial heterogeneity of the spectrum [1].
